# The Promising B−Type Response Regulator *hst1* Gene Provides Multiple High Temperature and Drought Stress Tolerance in Rice

**DOI:** 10.3390/ijms25042385

**Published:** 2024-02-17

**Authors:** Ermelinda Maria Lopes Hornai, Murat Aycan, Toshiaki Mitsui

**Affiliations:** 1 Department of Life and Food Sciences, Graduate School of Science and Technology, Niigata University, Niigata 950-2181, Japan; 2 National Division of Research and Statistics, Timor-Leste Ministry of Agriculture, Fisheries and Forest, Dili 626, Timor-Leste; 3 Laboratory of Biochemistry, Faculty of Agriculture, Niigata University, Niigata 950-2181, Japan

**Keywords:** *OsRR22*, yield, seedling stage, reproductive stage, antioxidant enzyme, gene expression

## Abstract

High temperatures, drought, and salt stresses severely inhibit plant growth and production due to the effects of climate change. The Arabidopsis *ARR1*, *ARR10*, and *ARR12* genes were identified as negative salt and drought stress regulators. However, in rice, the tolerance capacity of the *hst1* gene, which is orthologous to the *ARR1*, *ARR10*, and *ARR12* genes, to drought and multiple high temperature and drought stresses remains unknown. At the seedling and reproductive stages, we investigated the drought (DS) high temperature (HT) and multiple high temperature and drought stress (HT+DS) tolerance capacity of the YNU31−2−4 (YNU) genotype, which carries the *hst1* gene, and its nearest genomic relative Sister Line (SL), which has a 99% identical genome without the *hst1* gene. At the seedling stage, YNU demonstrated greater growth, photosynthesis, antioxidant enzyme activity, and decreased ROS accumulation under multiple HT+DS conditions. The YNU genotype also demonstrated improved yield potential and grain quality due to higher antioxidant enzyme activity and lower ROS generation throughout the reproductive stage under multiple HT+DS settings. Furthermore, for the first time, we discovered that the B−type response regulator *hst1* gene controls ROS generation and antioxidant enzyme activities by regulating upstream and downstream genes to overcome yield reduction under multiple high temperatures and drought stress. This insight will help us to better understand the mechanisms of high temperature and drought stress tolerance in rice, as well as the evolution of tolerant crops that can survive increased salinity to provide food security during climate change.

## 1. Introduction

Global climate change (high temperature, drought, salinity, irregular precipitations) seriously directly or indirectly limits plant growth and reduces yields [1,2,3]. According to climate simulations, surface air temperatures on Earth may rise by 1.4–5.8 °C over the next few decades [4]. According to several studies, the optimum plant growth temperature is 25–28 °C [5]; therefore, additional rises in average temperature or elevated temperatures during critical phases of the crop can impact both growth and yield detrimentally [6]. Extreme meteorological situations will result from rising average temperatures, and the frequency and severity of extreme weather loss (drought) will rise [7]. Extremely high temperatures can exacerbate the effects of drought stress by accelerating soil evaporation and increasing plant transpiration through stomata opening [8]. High temperatures and drought are frequently highly correlated [9]. Numerous studies have demonstrated that drought and high temperatures negatively impact plant physiology and growth [10,11,12]. High−temperature stress increases the vapor pressure deficit (VPD), leading to stomata closing and reducing CO_2_ delivery [13]. It may also disrupt photosynthetic apparatus and photochemistry, reducing photosynthetic rate and physiological reactions, such as chlorophyll loss and proline rise [14]. Drought stress reduces cell turgor and water content [15], restricting growth and dry mass formation. Plants constrict stomata to reduce water loss but also reduce photosynthesis [16]. Understanding how plants adapt to high temperatures and drought stress is necessary to highlight mechanisms and practices that should be developed to reduce the negative impacts [17], but in natural conditions, high temperatures and drought stress can often coexist.

Rice is an essential cereal crop consumed as a steady diet by half the world’s population; Asian countries supply more than 50% of rice production [18]. Aside from this, rice is extremely vulnerable to climate change because it requires optimal irrigation and a proper temperature to grow normally. It has been found that a 1 °C temperature increase might reduce paddy production by up to 10% [19,20]. In addition, drought stress is also the most yield−limiting factor in 35% of paddy fields worldwide, with rainfed regions experiencing 20–100% reductions in rice yield due to drought [21]. Decreasing plant productivity due to climate change and the food demand of the increasing world population seriously endangers food security. FAO projects that food production will need to rise by 70% by 2050 to meet the rising demand from the 2.4 billion population growth (10 billion) and dietary changes [22,23]. New high−yield, multiple stress−tolerant genotypes must be developed to meet the increasing demands of the global population and maintain food security [24].

Recently, some favorable genes were found to overcome multiple stress tolerances (salinity, drought, high temperature, and osmotic stress) in several species, such as Arabidopsis, beans, tomatoes, and wheat [25,26,27,28]. Although those genes offer crucial insights into multiple stress tolerance mechanisms, legal restrictions prevent these genetically modified plants from progressing beyond laboratory and/or greenhouse experiments [29]. Previously, a novel salt−tolerant HITOMEBORE SALT TOLERANT 1 (*hst1*) gene was developed [30]. The single nucleotide polymorphism (SNP) that conferred *hst1* its exceptional tolerance to salinity was found to be associated with the third exon of the RESPONSE REGULATOR 22 (*OsRR22*) in rice. *OsRR22* functions at the apex of a transcriptional cascade as a B−type response regulator (RR) protein. It possesses a C−terminal Myb−like DNA−binding domain and an N−terminal receiver domain, which confer specificity to the subsequent responses [31,32]. Recent research has suggested that the *A. thaliana ARR1*, *ARR10*, and *ARR12* genes, orthologous to *OsRR22* in rice, function as negative regulators of tolerance to drought [33] and salinity [34,35]. Although the *hst1* gene provides salinity tolerance in rice [35,36], Nahar et al. [37] highlighted that the YNU31−2−4 rice genotype carrying the *hst1* gene provided higher photosynthetic activity, lower Na^+^ ion accumulation, and high yield potential under multiple heat and salt stress. It was previously known that the *ARR1*, *ARR10*, and *ARR12* genes in Arabidopsis support the plants’ survival under drought stress [33]. However, the tolerance capacity of the *hst1* gene to drought and multiple high temperature + drought stresses is yet to be elucidated in rice. In this study, we examined the drought and multiple high temperature + drought stress tolerance capacity of the YNU31−2−4 (YNU) genotype, which carries the *hst1* gene, and its nearest genomic relative Sister Line (SL), which has a 99% identical genome without the *hst1* gene. For the first time, we found that the B−type response regulator *hst1* gene regulates the upstream and downstream genes to overcome yield reduction under multiple high temperatures and drought stress by controlling ROS accumulation and antioxidant enzyme activities. Our findings will help researchers understand how the *hst1* gene tolerates multiple HT+DS stress, as well as develop more stress−tolerant genotypes in the future.

## 2. Results

### 2.1. The Determination of Drought Tolerance Capacity at the Germination Stage

YNU (with *hst1*) and SL (without *hst1*) were examined for their ability to tolerate drought stress under 0, 5, 10, 15, 20, and 25% PEG6000 in vitro drought conditions to determine the minimal lethal dose (LD_50_). In our investigation, the LD_50_ was determined to be 15% PEG for all tested genotypes (Figure S3A). The plants continued to grow for seven days in both the control (0% PEG) and drought (15% PEG) conditions, although the shoot length of the YNU and SL genotypes decreased by 20 and 19%, respectively (Figure S3B). According to Figure S3C, the drought stress dramatically decreased root length in the YNU and SL genotypes by 29 and 30%, respectively.

### 2.2. The Effect of Drought, High Temperature, and Multiple High Temperature + Drought Stress on the Physiological Traits at the Seedling Stage

**Figure 1:**
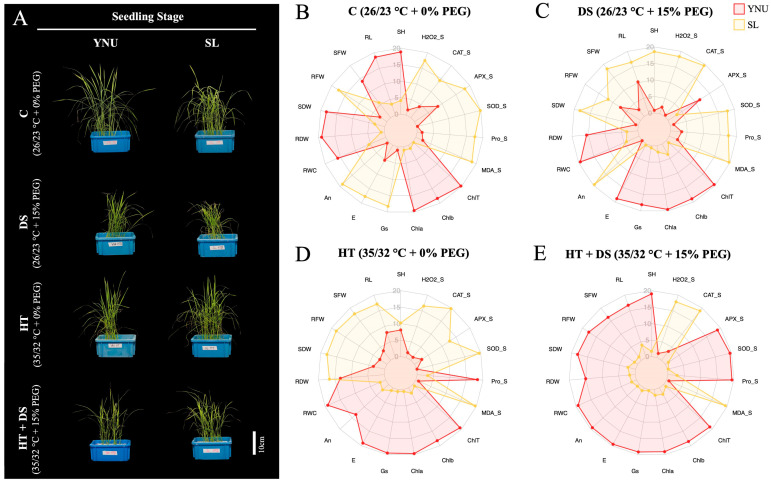
YNU genotype seedlings have increased chlorophyll and photosynthetic activity under high temperatures and drought stress. (**A**) The general view of stress affects genotypes at the seedling stage (24 DAG plants). The radar chart with normalized data of (**B**) control (C; 26/23 °C + 0% PEG), (**C**) drought (DS; 26/23 °C + 15% PEG), (**D**) high temperature (HT; 35/32 °C + 0% PEG), and multiple (**E**) high temperature + drought (HT+DS; 35/32 °C + 15% PEG) stress exposed YNU and SL genotypes. Shoot height (SH), root length (RL), shoot fresh weight (SFW), root fresh weight (RFW), shoot dry weight (SDW), root dry weight (RDW), relative water content (RWC), chlorophyll a (Chla), chlorophyll b (Chlb), total chlorophyll (ChlT) content, the net photosynthesis rate (An), transpiration rate (**E**), stomatal conductance (Gs), hydrogen peroxidase (H_2_O_2_), malondialdehyde (MDA), proline (PRO) content, superoxide dismutase (SOD), (**E**) catalase (CAT), ascorbate peroxidase (APX) activity, seedling stage (_S).

YNU and SL genotypes exposed to control (C; 26/23 °C–0% PEG), drought (DS; 26/23 °C–15% PEG) stress, high temperature (HT; 35/32 °C–0% PEG) stress, and multiple high temperature + drought (HT+DS; 35/32 °C–15% PEG) stress at the seedling stage are seen in Figure 1A. All stress treatments negatively affect measured physiological traits in YNU and SL genotypes at the seedling stages. Shoot height (SH) and root length (RL) were significantly reduced in YNU and SL under DS, HT, and multiple HT+DS conditions. The YNU showed higher SH and RL than SL under C and HT+DS conditions (Figure 1B,E and Figure S4A,B). Under DS, the SH of the SL genotype was found to be higher compared to the YNU genotype, but the RL of the SL genotype was recorded as almost the same (not significant differences) value as the YNU genotype (Figure 1C and Figure S4A,B). Although HT reduced SH and RL in both genotypes, SH and RL did not show significant differences among the YNU and SL genotypes (Figure 1D and Figure S4A,B). Shoot fresh weight (SFW) did not show significant differences between the YNU and SL genotypes under C conditions. The stresses reduced SFW in the YNU and SL genotypes. YNU showed significantly lower SFW than SL under DS and HT, but YNU showed higher SFW than SL under multiple HT+DS conditions (Figure 1B–E and Figure S4C). Root fresh weight (RFW) was recorded higher in the SL genotype under C and HT conditions than in the YNU genotype (Figure 1B,D and Figure S4D). Under DS, neither genotype’s RFW showed significant differences, but YNU showed significantly higher RFW than the SL genotype under HT+DS conditions (Figure 1C,E and Figure S4D). Shoot dry weight (SDW) was higher in the YNU genotype under C and HT+DS conditions than in the SL genotype (Figure 1B,E and Figure S4E). However, the SL genotype showed higher SDW under DS and HT stress conditions (Figure 1C,D and Figure S4F). Relative water content (RWC) did not show significant differences between YNU and SL genotypes under C conditions, but YNU showed higher RWC under DS, HT, and HT+DS conditions (Figure 1B–E and Figure S4G).

### 2.3. YNU Genotype Has Higher Chlorophyll Content and Photosynthesis Activity under Multiple High Temperatures and Drought Stress at the Seedling Stage

The chlorophyll a, b (Chla, Chlb), and total chlorophyll (ChlT) contents were lower in SL than in the YNU genotype under C and all stress conditions. Surprisingly, DS and HT+DS conditions lowered Chla, Chlb, and ChlT contents in both genotypes compared to C conditions. However, HT stress increased Chla, Chlb, and ChlT contents in both genotypes compared to C conditions (Figure S5A–C). The net photosynthesis rate (A_n_) was higher in the SL under C and DS conditions, but under HT and HT+DS conditions, YNU showed a higher A_n_ than the SL genotype (Figure 1B–E and Figure S5D). Transpiration rate (E) significantly increased in both genotypes under HT and HT+DS conditions but decreased under DS conditions compared to C conditions. The E was higher in the SL genotype under C conditions and did not show significant differences between YNU and SL under DS conditions. However, YNU showed higher E under HT and HT+DS conditions than the SL genotype (Figure 1B–E and Figure S5E). The stomatal conductance (g_s_) trait showed a similar pattern to the E trait among stress conditions and genotypes. The g_s_ were found only higher in SL under C conditions, but under DS, HT, and HT+DS conditions, g_s_ were detected higher in the YNU genotype (Figure 1B–E and Figure S5F).

### 2.4. YNU Genotype Showed Higher Antioxidant Enzymes and Osmoprotectants under Multiple High Temperatures and Drought Stress at the Seedling Stage

To understand the biochemical reflection of YNU and SL genotypes to C, DS, HT, and multiple HT+DS conditions, we analyzed hydrogen peroxidase (H_2_O_2_), malondialdehyde (MDA), proline content, superoxide dismutase (SOD), catalase (CAT), and ascorbate peroxidase (APX) activity at the seedling stage. All stress conditions increased H_2_O_2_ production in the YNU and SL plant tissues, but it was observed to be higher in the SL genotype even under the C condition compared to the YNU genotype. The H_2_O_2_ content of the SL genotype was higher under all stress conditions (Figure 1B–E and Figure S6A). The MDA level was increased by DS and multiple HT+DS stresses in both genotypes. Surprisingly, HT reduces the MDA level of YNU and SL genotypes. The SL genotypes showed higher MDA levels than the YNU genotype under all conditions (Figure 1B–E and Figure S6B). The proline (PRO) content was higher in the SL genotype than in the YNU genotype under C and DS conditions. Under HT and multiple HT+DS conditions, the YNU genotype showed higher PRO content than the SL genotype. While DS reduced PRO content compared to C, HT and multiple HT+DS stress increased PRO content in both genotypes (Figure 1B−E and Figure S6C). The SOD activity was found to be higher in SL genotype under C, DS, and HT conditions, but it was recorded to be higher in YNU genotype under multiple HT+DS conditions (Figure 1B–E and Figure S6D). Surprisingly, CAT activity was detected higher in the SL genotype than the YNU genotype under all C and stress conditions, and all stress conditions increased CAT activity in both genotypes (Figure 1B–E and Figure S6E). The APX activity was reduced in YNU and SL genotypes under stress conditions compared to C conditions, with one exception: YNU did not show significant differences among the APX activity of C and HT+DS conditions. The APX activity was recorded higher in the SL genotype than in the YNU genotype under C conditions. It did not show significant differences among SL and YNU genotypes under DS and HT conditions. However, the YNU genotype showed higher APX activity than the SL genotype under multiple HT+DS conditions (Figure 1B–E and Figure S6F). 

### 2.5. Multiple Stress Tolerance Supported by Higher Antioxidant Enzyme Activity and Osmoprotectant Production in the YNU Genotype at the Reproductive Stage

**Figure 2:**
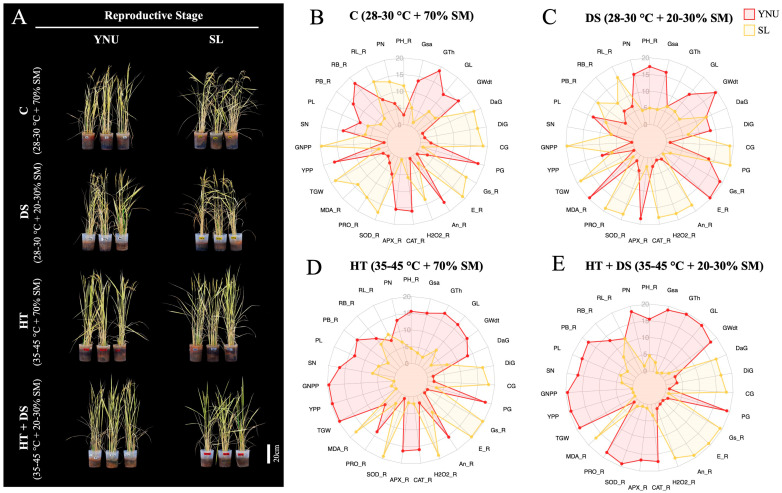
YNU genotype had higher yield and grain quality under HT and multiple HT+DS stress. (**A**) The general view of stress affects genotypes at the reproductive stage. The radar chart with normalized data of (**B**) control (C; 28–30 °C + 70% SM), (**C**) drought (DS; 28–30 °C + 20–30% SM), (**D**) high temperature (HT; 35–45 °C + 70% SM), and multiple (**E**) high temperature + drought (HT+DS; 35–45 °C + 20–30% SM) stress exposed YNU and SL genotypes. Soil moisture (SM), plant height (PH), root length (RL), plant biomass (PB), root biomass (RB), net photosynthesis rate (An), transpiration rate (E), stomatal conductance (Gs), hydrogen peroxidase (H_2_O_2_), malondialdehyde (MDA), proline (PRO) content, superoxide dismutase (SOD), catalase (CAT), ascorbate peroxidase (APX), panicle number (PN), panicle length (PL), spikelet number (SN), grain number per panicle (GNPP), yield per plant (YPP), 1000−grain weights (TGW), grain surface area (Gsa), grain thickness (Gth), grain length (GL), grain width (GWdt), damaged grain (DaG), die grain (DiG), chalky grain (CG), perfect grain (PG) percentage, reproductive stage (_R).

To test C, DS, HT, and HT+DS stresses on the reproductive stage of YNU and SL genotypes, 42-day-old plants were exposed to C, DS, HT, and HT+DS for two months. After harvesting, the plants` tolerance capacity was evaluated by several traits. The phenotype of YNU and SL genotypes after stress is seen in Figure 2A. All stresses significantly reduced plant height (PH) and root length (RL) in YNU and SL genotypes, but RH and RL traits did not show significant differences among genotypes under the same stress conditions (Figure 2B–E and Figure S7A,B). Stress factors significantly decreased plant biomass (PB) and root biomass (RB) traits. In particular, HT+DS dramatically reduced PB and RB in both genotypes compared to HT and DS. YNU genotype showed higher PB than SL under HT+DS condition (Figure 2B–E and Figure S7B,C). The A_n_ was higher in YNU under C and HT, but it was recorded higher in SL under DS and HT+DS conditions. Surprisingly, E and g_s_ were higher in the YNU genotype only under the DS condition, and SL showed higher E and g_s_ under C, HT, and HT+DS conditions (Figure 2B–E and Figure S7E–G).

After 38 days of stress exposure, multiple HT+DS increased H_2_O_2_ levels in both genotypes compared to the C condition. DS reduced H_2_O_2_ production in both genotypes, but the SL genotype showed higher H_2_O_2_ content than the YNU genotype under the C conditions. YNU showed lower H_2_O_2_ production than SL under C, HT, and multiple HT+DS stress conditions (Figure 2B–E and Figure S8A). The MDA content was significantly increased in both genotypes by stress application. The MDA level was higher in the SL genotype than in YNU under C, DS, HT, and multiple HT+DS conditions (Figure 2B–E and Figure S8B). The PRO concentration significantly decreased in the SL genotype by stress application. It was higher in the SL than in the YNU genotype under the C condition, but proline was lower in the SL genotype than the YNU genotype under stress conditions (Figure 2B–E and Figure S8C). The SOD activity increased in the YNU genotype under DS and multiple HT+DS conditions, and it was increased in the SL genotype only under HT conditions. The SOD activity was recorded higher in the SL genotype than the YNU genotype under C and DS conditions, and SOD activity was found higher in the YNU genotype than the SL genotype under DS and multiple HT+DS conditions (Figure 2B–E and Figure S8D). The CAT activity was increased in DS and multiple HT+DS conditions for both genotypes compared to C conditions. Although CAT activity was recorded as insignificant in YNU and SL genotypes under C, YNU showed higher CAT activity in DS, HT, and multiple HT+DS conditions than the SL genotypes (Figure 2B–E and Figure S8E). The YNU genotype showed higher APX activity than the SL genotype under C and all stress conditions (Figure 2B–E and Figure S8F).

### 2.6. YNU Genotype Showed Higher Yield Potential and Grain Quality under HT and Multiple HT+DS Stress Conditions

For several stress effects on yield and grain quality traits, we analyzed harvested plants in the YNU genotype, which showed significantly higher panicle number (PN) under DS and multiple HT+DS conditions than the SL genotype. The genotypes did not show significantly different PN under C and HT conditions. The panicle length (PL) reduces under stress conditions but does not significantly change between YNU and SL genotypes (Figure 2B–E and Figure S9A,B). The spikelet number (SN) was reduced in both genotypes under all stress conditions compared to the C, but the YNU genotype showed higher SN than the SL genotype under HT and multiple HT+DS conditions (Figure 2B–E and Figure S9C). The grain number per plant (GNPP) was higher in the SL genotype under C and DS conditions, but HT and multiple HT+DS conditions dramatically reduced GNPP in the SL genotype. The YNU genotype showed higher GNPP than SL under HT and multiple HT+DS conditions (Figure 2B–E and Figure S9D). Although HT and multiple HT+DS conditions significantly reduced yield in both genotypes, the YNU genotype yield was significantly higher than that of the SL genotype under HT and multiple HT+DS conditions. A similar pattern was observed in 1000−grain weight (TGW) traits, and YNU showed higher TGW than the SL genotype under HT and multiple HT+DS conditions (Figure 2B–E and Figure S9E,F).

The grain size, grain surface area (Gsa), grain thickness (Gth), grain length (GL), and grain width (Gwdt) were significantly reduced by DS and HT+DS. Still, YNU showed bigger grain morphology than the SL genotype under multiple HT+DS conditions (Figure 2B–E and Figure S10A–D). The SL genotype showed higher damaged grain (DaG) and died grain (DiG) under C and multiple HT+DS conditions. Surprisingly, single DS did not show significant differences between the DaG and DiG percentages of YNU and SL genotypes (Figure 2B–E and Figure S10E,F). The YNU genotype showed a lower chalky grain (CG) and higher perfect grain (PG) percentage than the SL genotype under C and all stress conditions (Figure 2B–E and Figure S10G,H).

### 2.7. Principal Component Analysis (PCA) Highlights Differences in Stress Tolerance between the YNU and SL Genotypes

**Figure 3:**
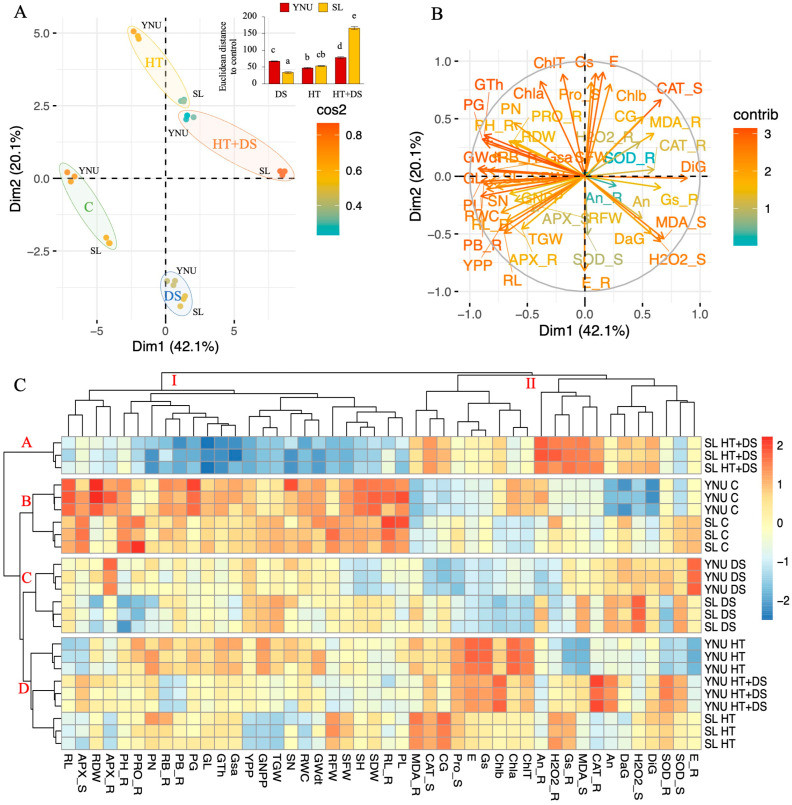
Principal component analysis (PCA) and hierarchical clustering analysis (HCA) highlight differences in stress tolerance between the YNU and SL genotypes. (**A**) principal component analysis (PCA), (**B**) PCA of the studied traits, (**C**) hierarchical clustering analysis of studied traits in YNU and SL genotypes under control (**C**), drought (DS), high temperature (HT), and multiple high temperature and drought (HT+DS) stress conditions at seedling stage (_S) and reproductive stage (_R). Means (±standard deviation) within the same graph followed by different letters are significantly different at *p* < 0.05 according to the Tukey HSD test from three independent biological replicates (*n* = 3). Shoot height (SH), root length (RL), shoot fresh weight (SFW), root fresh weight (RFW), shoot dry weight (SDW), root dry weight (RDW), relative water content (RWC), chlorophyll a (Chla), chlorophyll b (Chlb), total chlorophyll (ChlT) content, plant height (PH), root length (RL), plant biomass (PB), root biomass (RB), net photosynthesis rate (An), transpiration rate (E), stomatal conductance (Gs), hydrogen peroxidase (H_2_O_2_), malondialdehyde (MDA), proline (PRO) content, superoxide dismutase (SOD), catalase (CAT), ascorbate peroxidase (APX), panicle number (PN), panicle length (PL), spikelet number (SN), grain number per panicle (GNPP), yield per plant (YPP), 1000−grain weights (TGW), grain surface area (Gsa), grain thickenss (Gth), grain length (GL), grain width (GWdt), damaged grain (DaG), die grain (DiG), chalky grain (CG), perfect grain (PG) percentage.

All studied traits in the YNU and SL genotypes under C, DS, HT, and multiple HT+DS stress conditions at the seedling and reproductive stage were analyzed using principal component analysis (PCA) to evaluate the differentiation of genotypes using all data together. The PCA exhibits all analyzed data at the seedling and reproductive stage and variables associated with Dimmention1 (43.1%) and Dim2 (20.1%), of which Dim1 was the major component (total of 62.2%) (Figure 3A and Table S3). The colors of the separate variables indicate the quality of representation of the principal component, abbreviated as ‘Cos2’. The individuals are separated by genotype and condition. The green group indicates C, the blue group indicates DS, the yellow group indicates HT and the orange group indicates HT+DS conditions in the YNU and SL genotypes. The groups showed that responses to stress were different in both genotypes, but YNU showed better performance under HT+DS conditions (Figure 3A). The larger Euclidean distance between C and DS, HT, and HT+DS conditions was observed in the SL genotype under HT+DS conditions. Under the same conditions, YNU showed a lower Euclidean distance than the SL genotype. An opposite pattern was detected under DS conditions, and no significant differences between the Euclidean distance of YNU and SL genotypes were observed under HT (Figure 3A upper panel). The DiG, MDA_S, CAT_S, H2O2_S, Gs_R, CAT_R, MDA_R, DaG, CG, An, H2O2_R, An_R, Chlb, E, Gs, Pro_S, SOD_R, SOD_S traits were found higher under stress conditions in both genotypes compared to C conditions (Figure 3B).

The heatmap derived from a two−way hierarchical clustering analysis (HCA) indicated that all tested traits in YNU and SL genotypes under C and stress conditions were grouped into groups (Groups I and II). Group I represented the higher traits under C conditions, and Group II indicated the higher traits under DS, HT, and HT+DS conditions in both genotypes (Figure 3C). The genotypes (YNU and SL) under C and stress (DS, HT, and HT+DS) conditions were clustered into four clusters (Cluster A, B, C, and D). Cluster A indicated SL genotype under multiple HT+DS; Cluster B pointed to the C condition in both genotypes; Cluster C showed DS condition in both genotypes, and Cluster D represented HT condition in both genotypes and HT+DS condition in the YNU genotypes. The HCA indicated that the YNU genotype had higher growth performance and biochemical activity than the SL genotype against HT+DS stress (Figure 3C).

### 2.8. The hst1 Controls Multiple HT+DS Stress Tolerance by Regulating up and Downstream Genes

**Figure 4:**
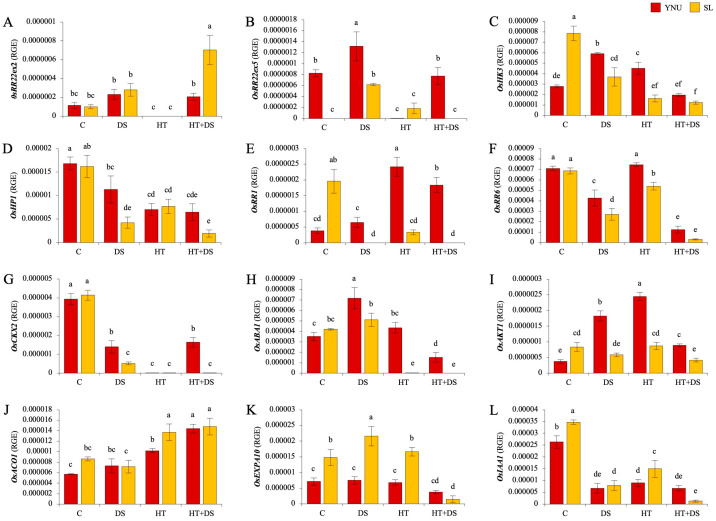
The *hst1* gene regulates several stress tolerance mechanisms by controlling the expression of genes both upstream and downstream. Relative gene expression of (**A**) *OsRR22*ex2, (**B**) *OsRRR22*ex5, (**C**) *OsHK3*, (**D**) *OsHP1*, (**E**) *OsRR1*, (**F**) *OsRR6*, (**G**) *OsCKX2*, (**H**) *OsABA1*, (**I**) *OsAKT1*, (**J**) *OsACO1*, (**K**) *OsEXPA10*, (**L**) *OsIAA1* genes under control (C), drought (DS), high temperature (HT), and multiple high temperature + drought (HT+DS) stress exposed YNU and SL genotypes. The reference gene *18sRNA* was used to standardize the Cq value for each sample. Means (±standard deviation) within the same graph followed by different letters are significantly different at *p* < 0.05 according to the Tukey HSD test from three independent biological replicates (*n* = 3).

The mutation resides in the third exon of the B−type response regulator 22 (Os06g0183100–*OsRR22*) gene (hitomebore salt tolerant 1–*hst1*). To examine any changes in the gene expression before and after the mutation point, we check relative gene expression (RGE) in the *OsRR22*ex2 and *OsRR22*ex5 genes. The DS condition significantly upregulated *OsRR22*ex2 gene expression, but no expression level was detected under HT stress conditions. Under HT+DS conditions, the expression level of *OsRR22*ex2 was increased in both genotypes, but the SL genotype showed higher expression than the YNU genotype (Figure 4A). After the mutation, the expression level of the OsRR22ex5 gene was not detected in the SL genotype under C and HT+DS conditions. While the DS condition increased RGE in both genotypes, HT stress significantly reduced RGE (Figure 4B). Previously, we detected the histidine kinase 3 (Os01g0923700–*OsHK3*) and histidine phosphotransfer 1 (Os08g0557700–*OsHP1*) genes controlled *hst1* gene as upstream genes. Here, we observed that SL has higher *OsHK3* gene expression under C conditions than the YNU genotype, but stress significantly reduces *OsHK3* gene expression in the SL genotype and significantly increases gene *OsHK3* expression in the YNU genotype. The YNU genotype showed a higher expression level of *OsHK3* under stress conditions than the SL genotype (Figure 4C). The expression of another upstream gene, *OsHP1*, was significantly decreased by stress applications. Under DS and HT+DS conditions, YNU showed significantly higher gene expression of *OsHP1* than the SL genotype (Figure 4D). 

The downstream genes of *hst1* genes, A−type response regulator 1 (Os04g0442300–*OsRR1*) and A−type response regulator 6 (Os04g0673300–*OsRR6*), showed different expression patterns in YNU and SL genotypes. The relative expression of the *OsRR1* gene was found to be higher in the SL genotype under C conditions, but it was reduced under HT and not detected under DS and HT+DS conditions. Expression of the *OsRR1* gene did not change under DS compared to C but significantly increased under HT and HT+DS conditions in the YNU genotype (Figure 4E). The *OsRR6* was reduced by DS and HT+DS conditions compared to C. Under C conditions, expression of the *OsRR6* gene did not show significant differences between YNU and SL genotypes, but under stress conditions, YNU showed higher expression of the *OsRR6* gene than the SL genotype (Figure 4F). The downstream gene expression of cytokinin−related (Os01g0197700–*OsCKX2*) was significantly reduced by stress application in both genotypes. The expression of *OsCKX2* was higher in the YNU genotype under DS and HT+DS conditions (Figure 4G). DS increased the abscisic acid−related (Os04g0448900–*OsABA1*) gene expression, but it was reduced by HT and HT+DS conditions. The YNU genotype showed higher expression of the *OsABA1* gene than the SL genotype under stress conditions (Figure 4H). The ion uptake−related (Os01g0648000–*OsAKT1*) gene expression increased in the YNU genotype under DS and HT conditions, and it was found higher in the YNU genotype than the SL genotype under stress conditions (Figure 4I). The ethylene−related (Os09g0451000–*OsACO1*) gene expression did not change under DS and HT+DS conditions. Under C and HT, it was higher in the SL genotype (Figure 4J). Stress applications in the SL genotype increased the cell elongation−related (Os04g0583500–*OsEXPA10*) gene expressions. The YNU genotype showed lower *OsEXPA10* expression than the SL genotype under C and stress conditions (Figure 4K). The IAA/Auxin−related (Os01g0178500–*OsIAA1*) was significantly reduced by stress application in both genotypes. The transcript level of the *OsIAA1* gene was found to be high in YNU only under HT+DS conditions (Figure 4L).

**Figure 5:**
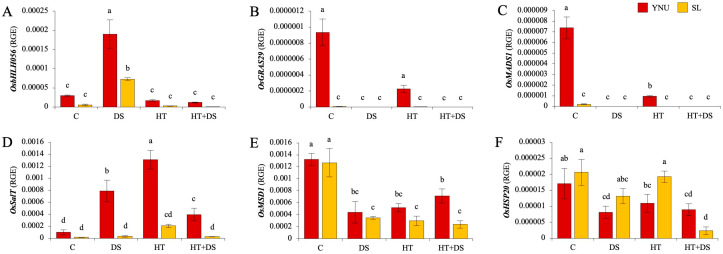
The transcription factors and stress−related genes that control *hst1* gene expression. Relative gene expression of (**A**) *OsbHLH056*, (**B**) *OsGRAS29*, (**C**) *OsMADS1*, (**D**) *OsSalT*, (**E**) *OsMSD1*, and (**F**) *OsHSP20* genes under control (C), drought (DS), high temperature (HT), and multiple high temperature + drought (HT+DS) stress exposed YNU and SL genotypes. The Cq values formed the basis of qRT−PCR. The reference gene *18sRNA* was used to standardize the Cq value for each sample. Means (± standard deviation) within the same graph followed by different letters are significantly different at *p* < 0.05 according to the Tukey HSD test from three independent biological replicates (*n* = 3).

The transcription factors and stress−related genes which control *hst1* gene expression such as *OsbHLH056* (Os01g0952800), *OsGRAS29* (Os05g0398800), *OsMADS1* (Os03g0215400), *OsSalT* (Os01g0348900), *OsMSD1* (Os05g0323900), and *OsHSP20* (Os03g0267000) genes were analyzed under C, DS, HT, and HT+DS conditions. The expression of the *OsbHLH056* gene was significantly increased in both genotypes under DS, and the *OsbHLH056* gene expression level was found to be higher in the YNU genotype than the SL genotype under C and stress conditions (Figure 5A). The *OsGRAS29* and *OsMADS1* genes were only expressed in YNU and SL genotypes under C and HT conditions, and expression level was detected higher in YNU genotype than SL genotype (Figure 5B,C). The transcript level of the *OsSalT* gene was significantly increased in the YNU genotype under stress conditions, and YNU showed higher expression of the *OsSalT* gene than the SL genotype under C and all stress conditions (Figure 5D). The expression of the *OsMSD1* gene was significantly reduced in both genotype under stress conditions, and it was higher in YNU genotype under HT+DS conditions (Figure 5E). The *OsHSP20* gene expression was detected lower in YNU and SL genotypes under stress conditions than the C condition. While SL showed higher *OsHSP20* gene expression under DS and HT conditions, YNU showed higher expression only under HT+DS conditions (Figure 4F). 

## 3. Discussion

The high temperature and drought stress commonly imbalance water usage of plants, from reducing photosynthesis to protein biosynthesis and formation damage [38,39]. The effectiveness of heat and drought stresses depends on the plant’s growth stages. The seedling stage of plants is generally assumed to be the most stress−sensitive stage for survival [40], but the reproductive stage stress exposure results in dramatic yield reductions [38]. In this study, we applied the HT, DS, and multiple HT+DS at separated seedling and reproductive stages to check *hst1* gene tolerance capacity. The YNU genotype with the *hst1* gene and the SL genotype without the *hst1* gene showed different growth patterns under C, DS, HT, and HT+DS conditions at the seedling stage. Although stresses reduced plant growth parameters such as SH, RL, SFW, RFW, SDM, and RDM, the YNU genotype showed better growth performance under multiple HT+DS conditions at the seedling stage. The RWC capacity of the YNU genotype was especially found to be significantly higher under all applied stress conditions. It shows the better tolerance of the YNU genotype under HT and DS stresses because those stresses can lead to loss of cell turgor and lower water content in plants [15]. This water loss leads to chlorophyll degradation and reduces photosynthesis [41]. DS especially degrades chlorophyll content in our experiment, but the YNU genotype promotes producing more chlorophyll than the SL genotype. This shows that the YNU genotype maintains a relatively used light energy more efficiently [42]. Higher chlorophyll content generally absorbs more energy and increases the photosynthesis rate; nevertheless, several studies have demonstrated that low chlorophyll concentrations do not always result in less photosynthesis [43,44]. Stress−reduced chlorophyll content did not reduce A_n_ in our experiment; the An increased at the seedling and reproductive stage under stress conditions. Especially under multiple HT+DS conditions, the YNU genotype promotes the A_n_ in the YNU genotype. A similar result was found by Aycan et al. [35] that the YNU genotype with the *hst1* gene supports the photosynthesis rate also under salt stress conditions.

The stress signals cause stomatal closure in leaves, causing the plant to conserve water and reduce the transpiration rate [45]. In our experiment, DS reduces the transpiration rate, but the transpiration rate (E) significantly increases with the HT. At the seedling stage, the YNU genotype showed a higher transpiration rate under stress conditions, but the opposite was recorded at the reproductive stage. A similar pattern was also observed in stomatal conductance (g_s_). By modulating stomatal opening, plants can reduce transpiration flux and water loss while restricting CO_2_ entry. It has been observed in previous studies that HT and DS increase transpiration and stomatal conductance [46]. This directly and indirectly increased ROS production by stressed plants [47]. Under stress conditions, H_2_O_2_ production was increased in both genotypes, but the *hst1* gene prevented the H_2_O_2_ production in the YNU genotype compared to the SL genotype. Overproduction of ROS triggers lipid peroxidation and cell damage [48]. The MDA level was significantly increased under stress conditions, and the YNU genotype exhibited less lipid peroxidation than the SL genotype. The lower production of ROS by the effect of the *hst1* gene resulted in less MDA production. Previously, under salinity and multiple salinity and high−temperature stress, the YNU genotype with the *hst1* gene showed a similar pattern [35,49]. 

To manage the elevated reactive oxygen species (ROS), plants possess several osmoprotectants and antioxidants that work together as a highly effective cooperative system [47]. Proline is one of the osmoprotectants that plays a vital role in plant stress tolerance. We found that stress conditions increased proline accumulation, and the *hst1* gene promoted PRO accumulation in the YNU genotype under multiple HT+DS stress conditions. Previous studies suggested that PRO accumulation increased under HT and DS [50,51]. The SOD, CAT, and APX are key ROS−scavenging enzymes that protect plant cells from excessive H_2_O_2_ damage [52]. Although the YNU genotype has lower SOD activity than SL under C conditions, under multiple HT+DS condition *hst1* gene may promote the SOD activity in the YNU genotype. Surprisingly, under HT, YNU has lower SOD activity than the SL genotype. YNU has lower CAT activity at the seedling stage, but the reproductive stage activity of CAT in the YNU genotype is higher than in the SL genotype. Additionally, stress conditions increased the CAT activity. Conversely, APX activity was lowered under stress conditions, but the *hst1* gene may still promote the APX activity in YNU under multiple HT+DS stress conditions. Previous studies reported similar results under HT and DS conditions [53,54,55]. The *hst1* gene promoted an antioxidant system in previous studies under salt stress and high−temperature stress conditions [35,37,49]. It demonstrated that increased PRO antioxidant enzyme activity may minimize ROS production under multiple HT+DS conditions [54,56]. The higher PRO and antioxidant production can be related to the regulatory mechanism of the *hst1* gene, which regularly controls the cytokinin signal mechanism in the cell [57,58,59].

Plants experiencing multiple HT and DS exhibit diminished growth, hastened senescence, and premature mortality compared to plants subjected to only one stressor [60]. Hence, the duration of the combined occurrence of DS and HT stress plays a vital role in determining the subsequent interaction effect [61]. This is because severe harmful consequences involving irreversible alterations emerge once a particular tipping point is reached. The non−linear relationship between stress duration and response complicates the prediction of combined stress impacts [62]. We applied the HT and DS a month before flowering to see the effect on the yield. The stresses did not affect panicle numbers but significantly reduced panicle length and spikelet number. The SL genotype showed higher GNPP under C and DS conditions, and YNU showed higher GNPP under HT and multiple HT+DS conditions. The yield was evaluated higher in YNU under C conditions and the same in DS conditions. The HT and multiple HT+DS conditions significantly reduce YPP in both genotypes, but the YNU genotype showed higher yield performance than the SL genotype. The YNU genotype supports higher yield potential and TGW under HT and multiple HT+DS conditions. Previously, the YNU genotype with the *hst1* gene promoted higher yield under salt and high temperature + salt stress conditions [36,37]. Stresses affect yield and reduce grain quality and market value [63]. The quality of rice grain is categorized by the combination of physical and chemical characteristics. Grain appearance, color, size, and shape, chalkiness, whiteness, degree of milling, bulk density, foreign matter content, and moisture content are some physical characteristics, while amylose content of the endosperm, gelatinization temperature of the endosperm starch, and Na content are chemical characteristics [64]. The *hst1* gene may promote grain size and perfect grain percentage in the YNU genotype under multiple HT+DS conditions.

The *hst1* gene is a mutant form of the *OsRR22* gene, which several researchers have determined to be a salinity tolerance gene [30,34,35,36]. The *A. thaliana ARR1*, *ARR10*, and *ARR12* genes are orthologous to the *OsRR22* gene and negatively regulate salinity tolerance and DS [33]. This study’s morpho−physiological and yield results showed that the *hst1* gene supports multiple HT+DS tolerance. The function of the *hst1* gene to provide multiple HT+DS tolerance might be related to the regulation of up and downstream genes of the *hst1* gene and transcription factors that can control the *hst1* gene [32,33,35]. The stresses reduce upstream (*OsHK3* and *OsHP1*) of the *hst1* gene expression levels, but the YNU genotype showed higher gene expression than the SL genotype. The *OsHK3* gene has a function of ROS scavenging, salinity, and drought tolerance in rice [65,66]. The *OsHPs* positively regulate the cytokinin signaling pathway and play different roles in salinity and DS [67]. Two of the main functions of the *hst1* gene is the controlling cytokinin signaling pathway and hormone crosstalk, previously *OsRR1*, *OsRR6*, *OsCKX2*, *OsABA1*, *OsAKT1*, *OsACO1*, *OsEXPA10* and *OsIAA1* genes were determined as the downstream of the *hst1* gene. Our results suggested that *OsRR1* and *OsRR6* genes are related to HT and HT+DS tolerance by controlling the *hst1* gene, and the tolerant genotypes showed higher expression under stress conditions. Previously, the relation of A−type RR genes, such as *OsRR6*, and DS relations was determined [68,69,70,71]. The cytokinin−related *OsCKX2* gene is known to regulate salinity tolerance negatively [68], but in our study, we detected that the *OsCKX2* gene has a response to DS, and the tolerant rice genotype showed higher expression. The *ZmbZIP33* gene is highly homologous with the abscisic acid−related *OsABA1* gene and is strongly upregulated by DS, HT, and salinity stresses [72]. The *OsABA1* gene is upregulated under DS and HT stresses; importantly, the tolerant genotype showed higher *OsABA1* gene expression than sensitive rice. The *OsAKT1* is involved in salinity and drought tolerance [73,74]. The DS, HT, and multiple HT+DS conditions significantly upregulated ion uptake−related *OsAKT1* gene expression, and tolerant rice (YNU) showed higher gene expression than sensitive ones (SL). In previous studies, the ethylene−related *OsACO1* gene was down−regulated under HT and salt stress conditions [35,75]. Surprisingly, the relative gene expression of the *OsACO1* gene was significantly increased by HT and multiple HT+DS conditions. The cell elongation−related *OsEXPA10* gene was determined as cell elongation, biotic stress resistance, and negative regulation of salinity stress in rice [35,76,77]. Here, we found that the *OsEXPA10* gene negatively regulates abiotic stress conditions. The IAA/Auxin−related *OsIAA* genes are involved in salt and drought tolerance in rice [78]. The expression of the *OsIAA1* gene is reduced under stress conditions. Additionally, *OsSALT* and *OsMSD1* genes support HT and DS stress tolerance in the YNU genotype [79,80]. 

## 4. Materials and Methods

### 4.1. Plant Material and Experimental Design

The ‘YNU31−2−4’ and ‘YNU31−2−4 Sister line’ *Oryza sativa* ssp. *japonica* rice genotypes were used as plant material. In this experiment, the ‘YNU31−2−4’ (YNU) genotype is used as a salt−tolerant [36], and the ‘YNU31−2−4 Sister Line’ (SL) genotype is used as a salt−susceptible [35]. The seeds of genotypes were provided by the Laboratory of Biochemistry, Faculty of Agriculture, Niigata University, Japan.

In order to evaluate normal−temperature as control (C), high−temperature (HT), drought stress (DS) and combine high−temperature drought stress (HT+DS) tolerance of salt−tolerant YNU and salt−susceptible SL genotypes at seedling and reproductive stages, seeds were de−husked and surface sterilized using 5% sodium hypochlorite (Fujifilm, Tokyo, Japan) solution for 20 min and then rinsed three times (1 min each) with sterile distilled water [81]. Sterilized seeds were planted on the ½ Murashige and Skoog (MS) medium (Ducefa Biochemie, Haarlem, The Netherlands) at pH 5.8. The germination percentage (GP) under NT, HT, DS, and HT+DS conditions was assessed by using ½ MS medium, including 15% PEG6000 (Fujifilm, Tokyo, Japan) under C (26/23 °C) (Day/night), HT (35/32 °C), DS (26/23 °C + 15% PEG), and HT+DS (35/32 °C + 15% PEG) after three days of incubation in dark conditions when the developing radicle grew to a length of 2 mm. 

The rice seedlings were grown on the ½ MS medium for seven days in a controlled growth chamber under light intensity 350 μmol m^−2^ s^−1^ at 26/23 °C cycles and 70% relative humidity. At DAG7, healthy and uniform−looking seedlings were transferred to a hydroponic system by placing them into holes (1 plant/hole) on a Styrofoam seedling float device, and the emerging radicle was carefully inserted through the nylon mesh. The Styrofoam device was suspended on a tray filled with Yoshida solution at pH 5.0 [82]. The salt and/or heat stresses were imposed on 14-day-old rice seedlings by supplementing Yoshida’s medium with 15% PEG6000 (drought) and/or high temperatures (35/32 °C) for 7 days. 24-day-old plants were harvested for morpho−physiological analysis at the seedling stage (Figure S1A). 

For the reproductive stage testing, sterilized seeds were placed on ½ MS medium for seven days in a controlled growth chamber under light intensity 350 μmol m^−2^ s^−1^ at 26/23 °C cycles. At DAG7, healthy and uniform−looking seedlings were transferred to nursery culture soil (0.5 g N, 0.9 g P, and 0.5 g K/kg) and grew under a controlled growth chamber under light intensity 350 μmol m^−2^ s^−1^ at 26/23 °C cycles and 70% relative humidity. The 14-day-old rice seedlings were transplanted into 1L pots with rice nursery culture soil and placed in a semi−conventional greenhouse for four weeks. Forty−two days old plants (DAG42; four weeks before flowering), YNU and SL genotypes were exposed to control (C; 28–30 °C and 70% soil moisture), DS (28–30 °C and 20–30% soil moisture), HT (HT; 35–45 °C and 70% soil moisture), and HT+DS (35–45 °C and 20–30% soil moisture) for eight weeks. Four weeks after flowering, stress treatments were removed, and plants were turned to control conditions (C; 28–30 °C and 70% soil moisture) until harvesting. Plants at 120 days old were harvested (Figure S1B). The soil moisture was monitored at a depth of 15–20 cm with a HydraGO machine every three days for each pot, and a sensitive thermometer measured temperatures. The air temperature was recorded at midday using a digital thermometer (Figure S2A). The HydraGO performs soil measurements utilizing the impedance−based HydraProbe soil sensor technology, which measures the actual and “imaginary” dielectric permittivity and completely characterizes a standing wave’s radio frequency energy distribution in the soil (Figure S2B). The experiments were designed by completely randomized design (CRD), and four biological replications were used for each genotype and stress treatment (C, DS, HT, and HT+DS).

### 4.2. Leaf Gas Exchange Measurement and Photosynthetic Pigments

A portable photosynthetic LI−6400XL system (LI−6400−20, LiCor Biosciences, Lincoln, NE, USA) was used to examine leaf gas exchange measurements. To ensure maximum results and a leaf temperature of 28–35 °C, measurements of the net photosynthetic rate (A_n_) (µmol CO_2_ m^−2^ s^−1^), stomatal conductance (g_s_) (mol H_2_O m^−2^ s^−1^), and transpiration rate (E) (mmol H_2_O m^−2^ s^−1^) were made during the flowering stage on sunny, clear days from 9:00–12:00. Three leaves of rice plants were used to measure the leaf gas exchange for each treatment.

Chlorophyll contents were measured according to Grimme and Boardman [83], chlorophyll a, b, and total. 50 mg of fresh leaf material was exposed to 1.5 mL of methanol in the dark for two hours at 23 °C. Using a double−beam spectrophotometer U−2900 (Hitachi, Tokyo, Japan), the absorbances were measured at 650 and 665 nm. According to the following formula, the chlorophyll a (Chla), chlorophyll b (Chlb), and total chlorophyll (ChlT) contents were calculated.

Chla = (16.5 × A665 − 8.3 × A650) × volume (V)/weight (W),

Chlb = (33.8 × A650 − 812.5 × A665) × V/W,

ChlT= (4 × A655 + 25.5 × A650) × V/W.

### 4.3. Sampling, Phenotyping, and Harvesting Determination

The growth performance of the seedlings was evaluated by measuring the shoot height (SH), root length (RL), shoot fresh weight (SFW), root fresh weight (RFW), shoot dry matter (SDM), root dry matter (RDM) and relative water content (RWC). The RWC was estimated using the following formula by Sade et al. [84];

RWC = (FW − DM)/(Turgid Weight − DM) × 100.

The grains reached physiological maturity and the primary agronomic traits, including plant height (PH), root length (RL), plant biomass (PB), root biomass (RB), tiller number (TN), panicle number (PN), panicle length (PL), spikelet number (SN), 1000−grain weight (TGW), grain number per panicle (GNP), and yield per plant (YP), were measured. Three plants per genotype and treatment were used to determine the parameters. Perfect grain (PG), chalky grain (CG), grain whiteness (GW), grain length (GL), grain width (GWd), grain thickness (GTh), and grain surface area (GSFA) were determined using a rice grain grader (RGQI20A, Satake, Hiroshima, Japan).

### 4.4. Biochemical Analysis

Free proline content was determined according to the method described by Bates et al. [85], with a slight modification. Fresh leaf samples (0.5 g) were briefly homogenized in 10 mL of 3% sulfosalicylic acid and incubated for 24 h at 4 °C. The homogenate was centrifuged at 10,000× *g* at 25 °C for 5 min, and the supernatant (1 mL) was reacted with 1 mL of ninhydrin reagent and 1 mL of glacial acetic acid in a test tube at 100 °C for 1 h, and the reaction was stopped by submerging the tubes in an ice bath for 20 min. Proline was extracted with 2 mL of toluene and incubated at room temperature for 30 min. The toluene phase was discarded, and absorbance was read at 520 nm using Double Beam Spectrophotometer U−2900 (Hitachi, Tokyo, Japan). 

Malondialdehyde (MDA) determination was estimated according to an optimized method of Dhindsa and Matowe [86]. In brief, the leaf sample (0.5 g) was homogenized with 5 mL of 0.1% trichloroacetic acid and centrifuged at 12,500× *g* at 25 °C for 20 min. The supernatant (2 mL) was mixed with 2 mL thiobarbituric acid−TCA. The mixture was incubated for 30 min at 90 °C, and the reaction stopped by placing the tube in an ice bath for 10 min. The chromogen formed was measured at 520 and 600 nm using Double Beam Spectrophotometer U−2900 (Hitachi, Tokyo, Japan).

Frozen leaf powder samples (1 g) were homogenized in a cold mortar with 4 mL of 1M phosphate buffer (pH 7.0) containing 0.1 mM of Na−EDTA (10 mL). The homogenate was centrifuged at 18,000× *g* for 15 min at 4 °C, and the supernatant was used to measure antioxidant enzyme activities [87].

The hydrogen peroxide (H_2_O_2_) concentration was determined using Loreto and Velikova’s method (2001). A 0.3 g leaf sample was homogenized in 3 mL of 1% (*w*/*v*) trichloroacetic acid (TCA). The homogenate was centrifuged for 10 min at 10,000× *g* and 4 °C. The supernatant was then mixed with 0.75 mL of 10 mM K−phosphate buffer (pH 7.0) and 1.5 mL of 1 M KI. The supernatant’s H_2_O_2_ concentration was determined by comparing its absorbance at 390 nm to a standard calibration curve. A standard curve plotted in the range of 100 to 1000 mol/mL was used to calculate the concentration of H_2_O_2_. The concentration of H_2_O_2_ was expressed in µmol g^−1^ FW.

Superoxide dismutase (SOD) activity estimation was based on the ability of SOD to inhibit the photochemical reduction of nitro blue tetrazolium (NBT) and was assayed by the method of Cakmak and Marschner [88]. One unit of SOD was defined as the amount of enzyme required to induce a 50% inhibition of NBT reduction at 25 °C. The activity of SOD was expressed at unit min^–1^ g^–1^ of FW. The absorbance was read at 650nm using a Double Beam Spectrophotometer U−2900 (Hitachi, Tokyo, Japan).

Catalase (CAT) activity was determined by monitoring the decrease in absorbance at 240 nm for 3 min following the consumption of H_2_O_2_ [89]. The reaction mixture consisted of 0.8 mL of a 50 mM phosphate−buffered solution (pH 7.6) containing 0.1 mM Na−EDTA, 0.1 mL of 100 mM H_2_O_2_, and 0.1 mL of enzyme extract in a 2 mL volume.

Ascorbate peroxidase (APX) activity was assessed as a decrease in absorbance at 290 nm for 1 min according to the method of Amako et al. [90]. The assay solution contained 100 μL of extract sample, 50 mM potassium phosphate buffer (pH 7.6), 0.5 mM H_2_O_2_, and 0.1 mM ascorbate. The reaction was initiated by adding the enzyme extract, and the decrease in absorbance was recorded.

### 4.5. Real−Time Quantitative PCR Analysis

High−quality total RNA was extracted from leaf tissues of YNU and SL rice plants exposed to all stress conditions at seedling and reproductive stages employing the TRizol method [91] following the manufacturer’s instructions (Invitrogen, CA, USA). In Nucleic Acid mode, concentration and purity were assessed using the NanoDropTM One/OneC Microvolume UV−Vis Spectrophotometer (Thermo Fisher Scientific, Waltham, MA, USA). The cDNA templates were produced from the total RNA samples through reverse transcription, utilizing the ReverTra Ace^®^ qPCR RT Master Mix with gDNA Remover (Toyobo, Osaka, Japan).

The real−time polymerase chain reaction (RT−PCR) analysis was conducted using the CFX96^TM^ Real−Time PCR Detection System (Bio−Rad Laboratories GmbH, Hercules, CA, USA). The RT−PCR amplifications were conducted using a reaction volume of 10 μL. The reaction mixture consisted of 5 μL of SsoFastTM EvaGreen^®^ Supermix (Bio−Rad Laboratories GmbH, Hercules, CA, USA), 3.6 μL of ddH_2_O, 0.2 μL of 10 pmol sense (forward) primer, 0.2 μL of 10 pmol antisense (reverse) primer, and 1 μL of cDNA. The PCR technique was followed to perform triplicate reactions for each gene. The melting curve for the experiment consisted of the following temperature conditions: a 2 min at 98 °C, followed by a 2s at 98 °C, then a 5 s at 60 °C, and finally a 10 s ranging from 75 to 95 °C. This cycle was repeated 40 times. The normalization of gene expression for the target gene (Table S1) was conducted using the 2^−∆∆CT^ method as proposed by Livak and Schmittgen [92] employing reference gene *18SrRNA* (AF069218).

### 4.6. Data Analysis and Statistics

Recorded data was subjected to an analysis of variance (ANOVA) to determine genotype, temperature, and drought differences using R software (version 3.6.1, available at https://www.r−project.org/) and Genstat^®^ v.18 software (VSN International Ltd., Hertfordshire, UK). The ‘glht’ function in the’multcomp’ package of the R programming language was used to determine means separation using Tukey’s honestly significant difference (HSD) test at *p* < 0.05 [93]. 

Principal component analysis (PCA) was conducted on the correlation matrix of YNU and SL genotypes and C, HT, DS, and HT+DS treatments for comparison purposes. The index values for each treatment were initially determined by evaluating genotypes and treatments. The attributes within each treatment were aggregated and utilized as index values for conducting a Principal Component Analysis (PCA). The index values were employed to ascertain the correlation between response variable vectors and to make comparisons across the ordination space. The same dataset was also subjected to two−way heatmap clustering analysis (HCA). The Pearson correlation coefficient was employed as a distance metric based on correlation. The dissimilarity matrix was computed using the Euclidean technique. PCA and HCA analyses were conducted using the RStudio software (Version 2023.06.2+561), employing the ‘prcomp’ function from the ‘factoextra’ package [94]. The data underwent hierarchical clustering using the heatmap function in the ‘pheatmap’ package within the RStudio software [95].

## 5. Conclusions

**Figure 6:**
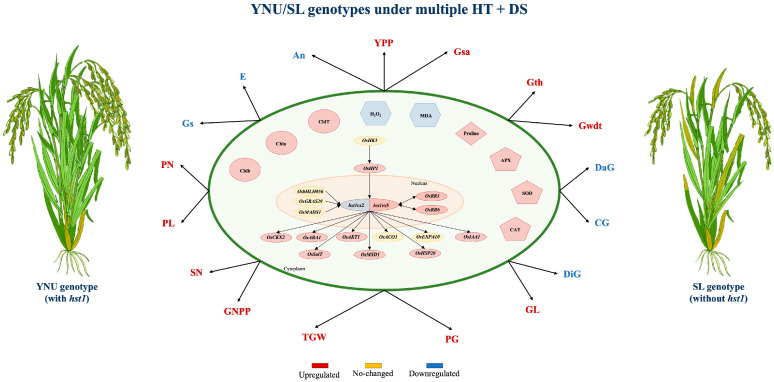
Suggested schematic illustration of a working model depicting the *hst1* regulation against multiple HT+DS stress at the reproductive stage. Chlorophyll a (Chla), chlorophyll b (Chlb), total chlorophyll (ChlT) content, net photosynthesis rate (An), transpiration rate (E), stomatal conductance (Gs), hydrogen peroxidase (H_2_O_2_), malondialdehyde (MDA), proline (PRO) content, superoxide dismutase (SOD), catalase (CAT), ascorbate peroxidase (APX), panicle number (PN), panicle length (PL), spikelet number (SN), grain number per panicle (GNPP), yield per plant (YPP), 1000−grain weights (TGW), grain surface area (Gsa), grain thickness (Gth), grain length (GL), grain width (GWdt), damaged grain (DaG), die grain (DiG), chalky grain (CG), perfect grain (PG) percentage.

Plant growth performance in all genotypes was reduced by stress scenarios applied during both the vegetative and reproductive stages. The findings indicated that the YNU (*hst1*) genotype exhibited enhanced resilience to elevated temperatures, drought, and other forms of stress during the early growth phase. This was attributed to elevated photosynthetic pigments and activities, accumulation of osmoprotectants, and improved antioxidant enzyme activities. The *OsRR1*, *OsABA1*, *OsAKT1*, and *OsSalT* genes enhance gene expression in YNU (*hst1*) when exposed to drought stress (DS), high temperature (HT), and the combination of high temperature and drought stress (HT+DS). During drought conditions, the SL genotype showed a 2% improvement in yield performance. In contrast, the YNU genotype exhibited a 60% increase in grain number per panicle and a 4% increase in thousand−grain weight under high−temperature conditions. Our data confirm that the *hst1* gene facilitates the ability to tolerate numerous stressors by regulating gene expression and managing reactive oxygen species (ROS) through the activity of antioxidant enzymes (Figure 6).

## Data Availability

Not applicable.

## Supplementary Materials

The following supporting information can be downloaded at: https://www.mdpi.com/article/10.3390/ijms25042385/s1.
