# Supplementary material for: The Promising B−Type Response Regulator hst1 Gene Provides Multiple High Temperature and Drought Stress Tolerance in Rice
Source: Int J Mol Sci. 2024 Feb 17;25(4):2385. doi: 10.3390/ijms25042385 (PMC10889171; doi:10.3390/ijms25042385)
Supplement: Supplementary file 1 [file ijms-25-02385-s001.zip › Figure S10.pdf]

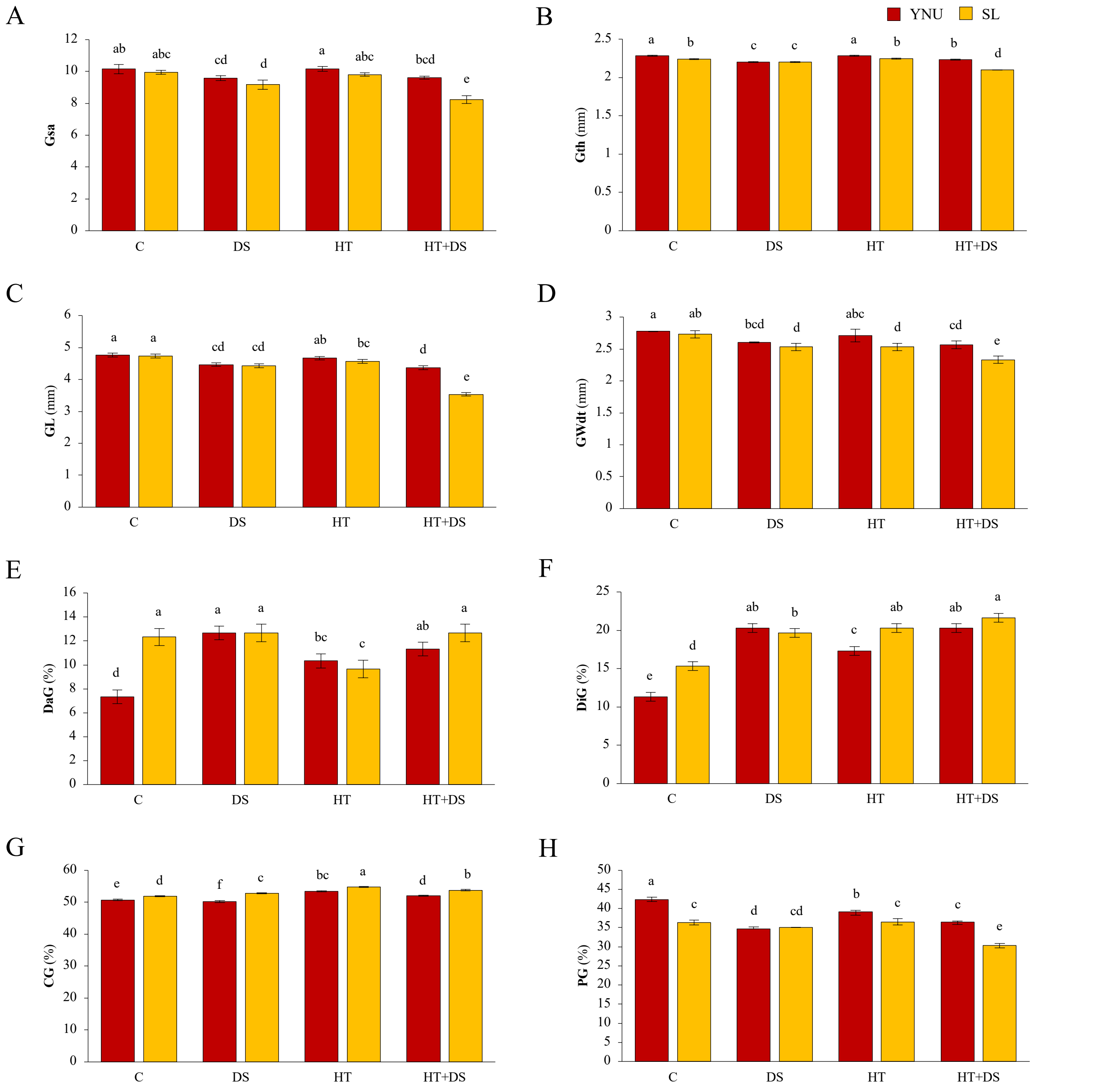

Figure S10. The effect of stresses on the grain quality traits of YNU and SL genotypes at the reproductive stage. **(A)** grain surface area (Gsa), **(B)** grain thickness (Gth), **(C)** grain length (GL), **(D)** grain width (GWdt), **(E)** damaged grain (DaG), **(F)** die grain (DiG), **(G)** chalky grain (CG), **(H)** perfect grain (PG) percentage of control (C; 26/23 ℃ + 0% PEG), drought (DS; 26/23 ℃ + 15% PEG), high temperature (HT; 35/32 ℃ + 0% PEG), and high temperature + drought (HT+DS; 35/32 ℃ + 15% PEG) stress exposed YNU and SL genotypes. Means (± standard deviation) within the same graph followed by different letters are significantly different at $p < 0.05$ according to the Tukey HSD test from three independent biological replicates (n=3).
