# Supplementary material for: The Promising B−Type Response Regulator hst1 Gene Provides Multiple High Temperature and Drought Stress Tolerance in Rice
Source: Int J Mol Sci. 2024 Feb 17;25(4):2385. doi: 10.3390/ijms25042385 (PMC10889171; doi:10.3390/ijms25042385)
Supplement: Supplementary file 1 [file ijms-25-02385-s001.zip › Figure S2.pdf]

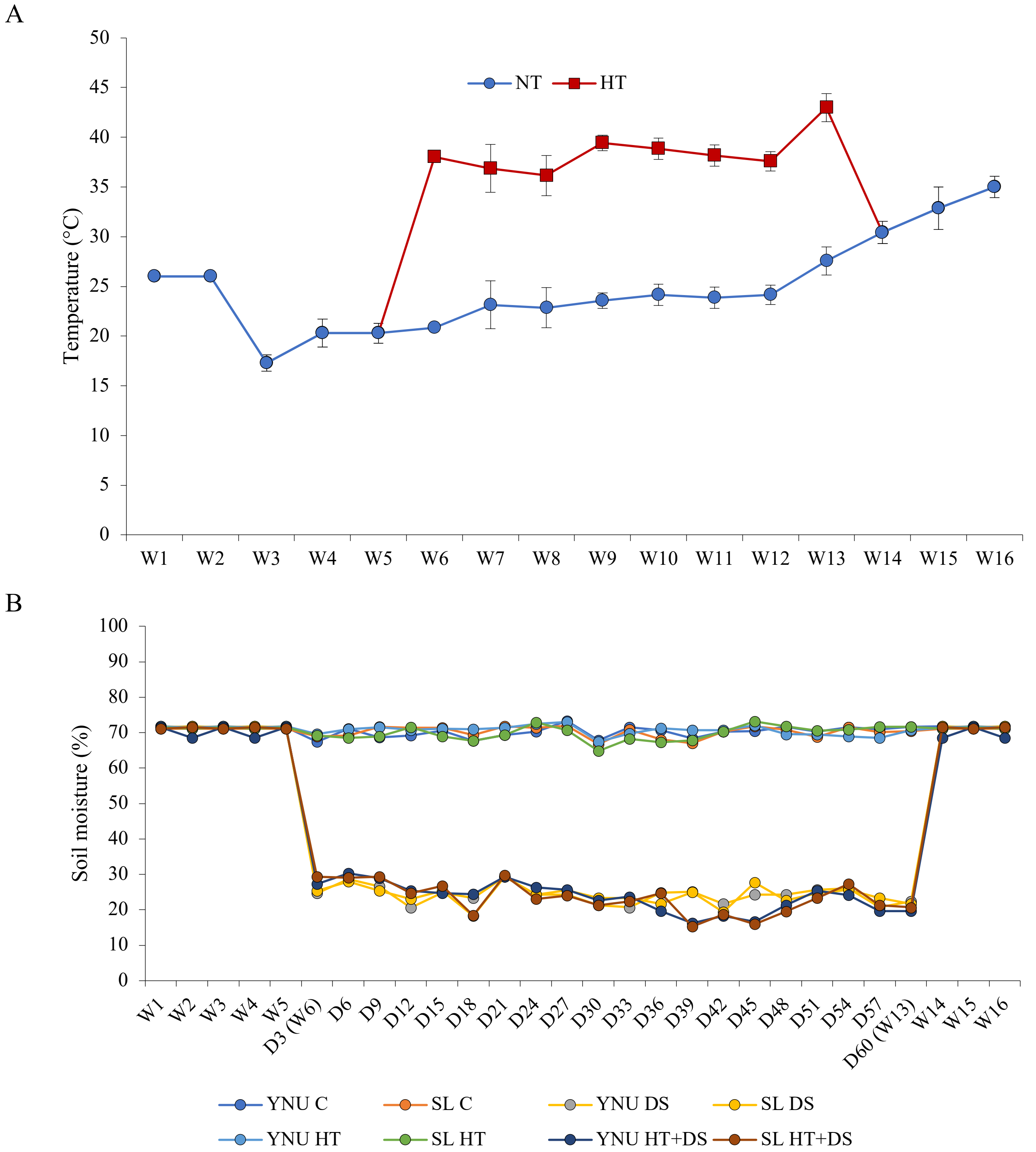

Figure S2. The greenhouse condition and drought application at the reproductive stage. **(A)** greenhouse temperature, **(B)** soil moisture percentage measurements.
