# Supplementary material for: The Promising B−Type Response Regulator hst1 Gene Provides Multiple High Temperature and Drought Stress Tolerance in Rice
Source: Int J Mol Sci. 2024 Feb 17;25(4):2385. doi: 10.3390/ijms25042385 (PMC10889171; doi:10.3390/ijms25042385)
Supplement: Supplementary file 1 [file ijms-25-02385-s001.zip › Figure S3.pdf]

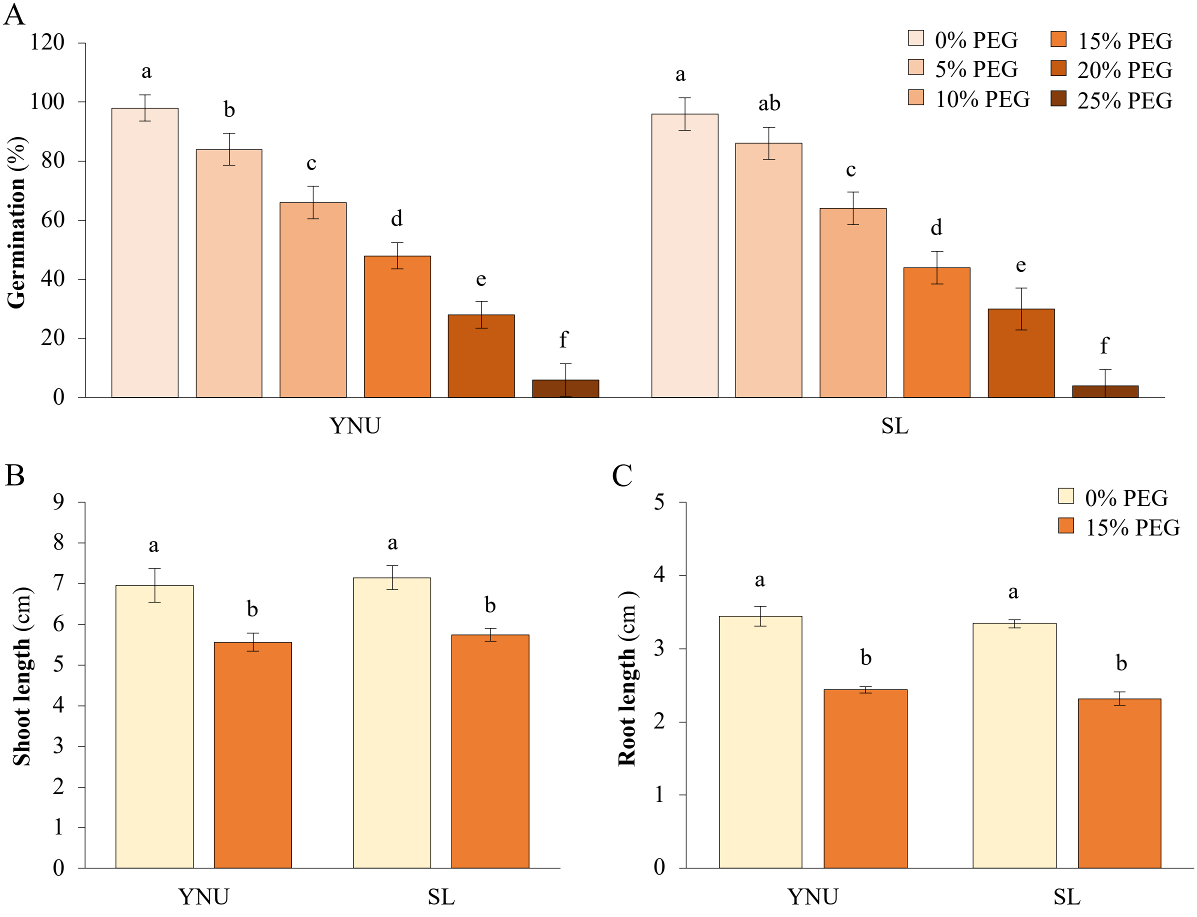

Figure S3. Drought stress determination. **(A)** Determination of LD_{50} germination for 3 days drought tolerance (PEG6000), **(B)** shoot length under 0 and 15% PEG, **(C)** root length under 0 and 15% PEG for 7 days in YNU and SL rice genotypes. Means (± standard deviation) within the same graph followed by different letters are significantly different at $p < 0.05$ according to the Tukey HSD test from three to five independent biological replicates (n=5).
