# Supplementary material for: The Promising B−Type Response Regulator hst1 Gene Provides Multiple High Temperature and Drought Stress Tolerance in Rice
Source: Int J Mol Sci. 2024 Feb 17;25(4):2385. doi: 10.3390/ijms25042385 (PMC10889171; doi:10.3390/ijms25042385)
Supplement: Supplementary file 1 [file ijms-25-02385-s001.zip › Figure S5.pdf]

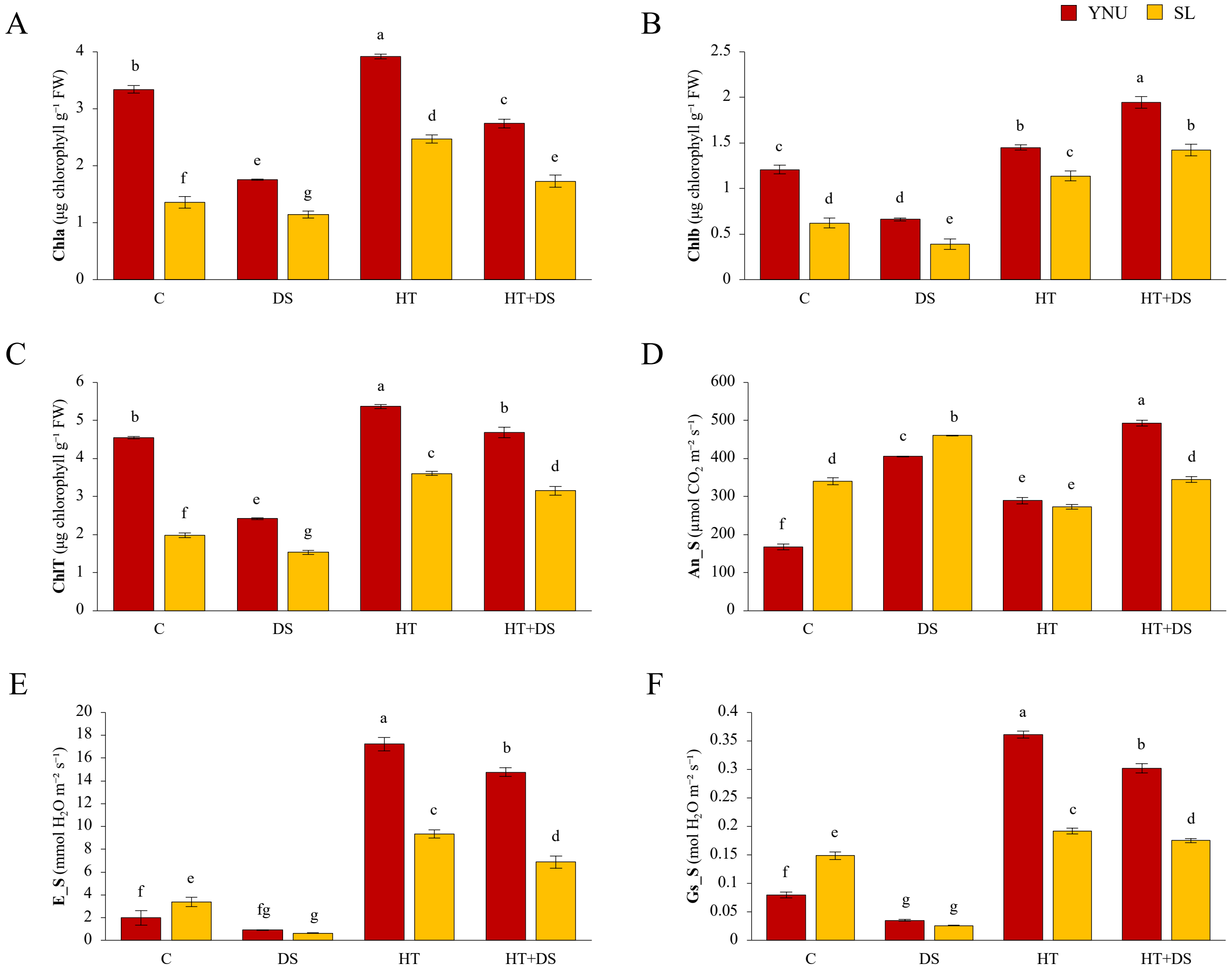

Figure S5. The effect of stresses on the chlorophyll and photosynthesis traits of YNU and SL genotypes at the seedling stage. **(A)** chlorophyll a (Chla), **(B)** chlorophyll b (Chlb), **(C)** total chlorophyll (ChlT) content, **(D)** the net photosynthesis rate (An), **(E)** transpiration rate (E), **(F)** stomatal conductance (Gs) of control (C; 26/23 °C + 0% PEG), drought (DS; 26/23 °C + 15% PEG), high temperature (HT; 35/32 °C + 0% PEG), and high temperature + drought (HT+DS; 35/32 °C + 15% PEG) stress exposed YNU and SL genotypes. Means (± standard deviation) within the same graph followed by different letters are significantly different at $p <$ 0.05 according to the Tukey HSD test from three independent biological replicates (n=3).
