# Supplementary material for: The Promising B−Type Response Regulator hst1 Gene Provides Multiple High Temperature and Drought Stress Tolerance in Rice
Source: Int J Mol Sci. 2024 Feb 17;25(4):2385. doi: 10.3390/ijms25042385 (PMC10889171; doi:10.3390/ijms25042385)
Supplement: Supplementary file 1 [file ijms-25-02385-s001.zip › Figure S9.pdf]

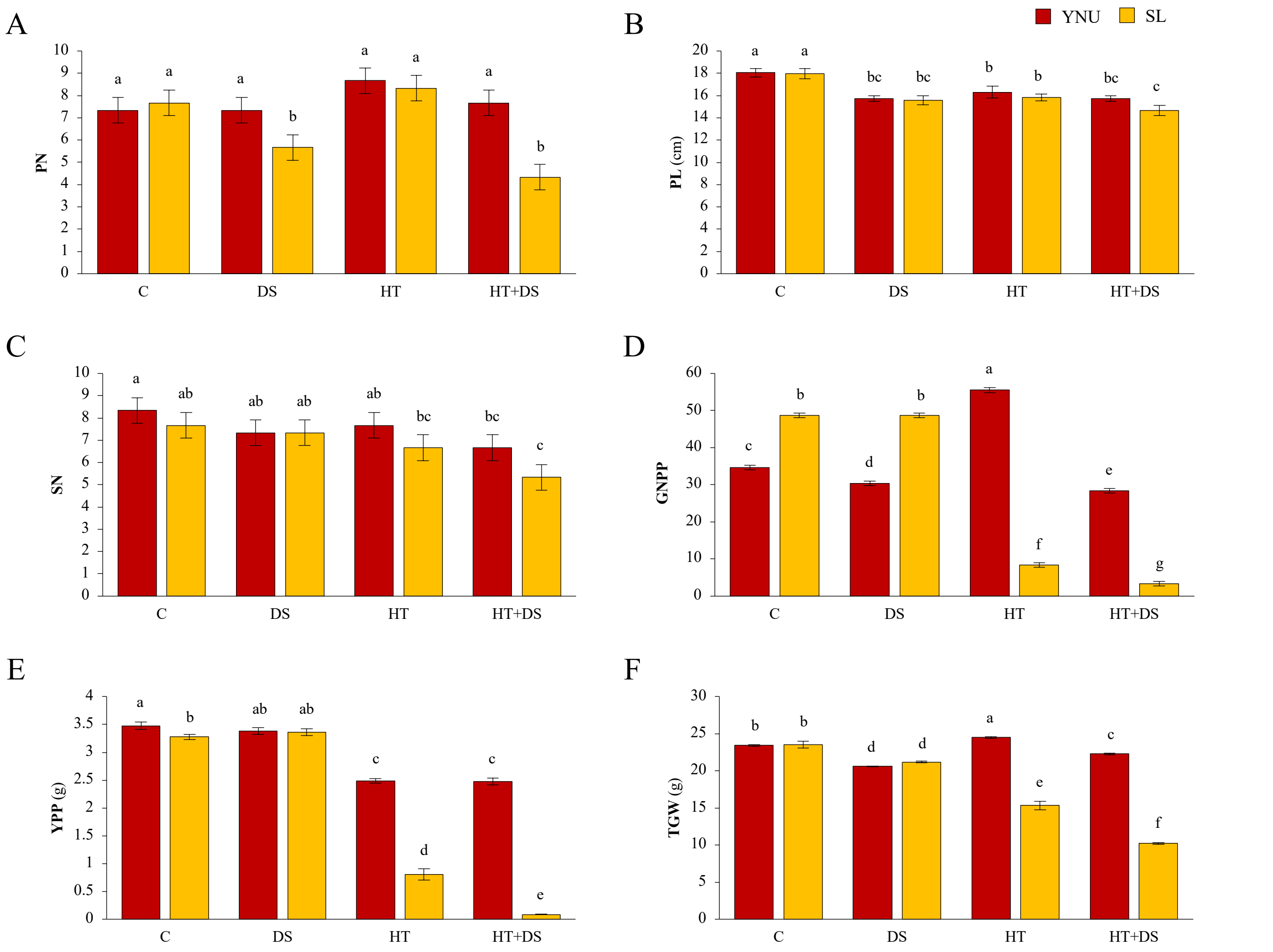


**Figure S9.** The effect of stresses on the harvest traits of YNU and SL genotypes at the reproductive stage. **(A)** panicle number (PN), **(B)** panicle length (PL), **(C)** spikelet number (SN), **(D)** grain number per panicle (GNPP), **(E)** yield per plant (YPP), **(F)** 1000-grain weights (TGW) of control (C; 28-30 °C + 70% PEG), drought (DS; 26/23 °C + 15% PEG ), high temperature (HT; 35/32 °C + 0% PEG), and high temperature + drought (HT+DS; 35/32 °C + 15% PEG) stress exposed YNU and SL genotypes. Means (± standard deviation) within the same graph followed by different letters are significantly different at $p < 0.05$ according to the Tukey HSD test from three independent biological replicates (n=3).
