# Supplementary material for: The Promising B−Type Response Regulator hst1 Gene Provides Multiple High Temperature and Drought Stress Tolerance in Rice
Source: Int J Mol Sci. 2024 Feb 17;25(4):2385. doi: 10.3390/ijms25042385 (PMC10889171; doi:10.3390/ijms25042385)
Supplement: Supplementary file 1 [file ijms-25-02385-s001.zip › Table S1.pdf]

Table S1. Sequence list of primers used in RT-PCR analysis

Gene name and ID	Primer name	Sequence
OsRR22 – hst1 (Os06g0183100)	*OsRR22* Ex2−F	CAGGCTATTACTGCGTTGAAG
	OsRR22 Ex2−R	GGAGCTTAAATCCGTCCATGT
	OsRR22 Ex5−F	CTTTCGGACTTCAGGAGCTTG
	OsRR22 Ex5−R	TCTGTTGAAGCTGAGGTTGC
OsCERK1 (Os08g0538300)	*OsCERK1*−F	CAACATGGCGGATCTCTTCG
	OsCERK1−R	GGACATCTGGAAGGGGAAGG
OsHK3 (Os01g0923700)	*OsHK3*−F	CATGTCAGCGTGCTCAACTT
	OsHK3−R	GATGACGTTATTGCCAGCCA
OsHP1 (Os08g0557700)	*OsHP1*−F	GAGGTCGTCACGCTCTTCT
	OsHP1−R	ACGAGTCCACCCTGTCAAAA
OsRR1 (Os04g0442300)	*OsRR1*−F	CTGAAGCCGATCCCAGTGA
	OsRR1−R	TGCTTTGAAGAGGCTTTACGA
OsRR6 (Os04g0673300)	*OsRR6*−F	ATCACCGACTACTGGATGCC
	OsRR6−R	TCTCCGACGACATGATCACC
OsCKX2 (Os01g0197700)	*OsCKX2*−F	CCGTCCTCATCTACCCCATG
	OsCKX2−R	ATCTCGTCGTTCTGCTCCTC
OsABA1 (Os04g0448900)	*OsABA1*−F	GATACAGATCCAGAGCAACGC
	OsABA1−R	ATAGCATCATCACCAACCGC
OsAKT1 (Os01g0648000)	*OsAKT1*−F	ATCCTTGTGCCAGTTGTTGC
	OsAKT1−R	TCCTTTACGACACCAGCCAT
OsEXPA10 (Os04g0583500)	*OsEXPA10*−F	AACCACCATTTCCTCCTCCC
	OsEXPA10−R	AGGACTGTCAGGAAGAAGCT
OsIAA1 (Os01g0178500)	*OsIAA1*−F	GGTGAGGTCGTTCAGGAAGA
	OsIAA1−R	CGGATGGTGAAGTGGGAGAA
OsACO1 (Os09g0451000)	*OsACO1*−F	GGCAGCATTGTCGTTCCC
	OsACO1−R	TTCTCCACCTCGTCCATCAG
OsGRAS29 (Os05g0398800)	*OsGRAS29*−F	TGGAGTTTCTGGAGAGAGGC
	OsGRAS29−R	GTTGGACGCCGTGAAGCC
OsMADS1 (Os03g0215400)	*OsMADS1*−F	ACAGGAAACCAGTGCAGAGA
	OsMADS1−R	GTCTGCTGCTTCATTGCTCA
OsbHLH056 (Os01g0952800)	*OsbHLH056*−F	GCGGGTTCAGTTCTTCCTCT
	OsbHLH056−R	GCTTTGCTTTGTTCCTGACG
OsSALT (Os01g0348900)	*OsSALT*−F	CGGTGTGGATGGACAGGAAT
	OsSALT−R	GGACTCCAGCCTCGTATGTA
OsMSD1 (Os05g0323900)	*OsMSD1*−F	AGGTGCTGCTTTACAAGGA
	OsMSD1−R	CCCAACAAAGGAACCAAGTT
OsHSP20 (Os03g0267000)	*OsHSP20*−F	AGGTGCTGCTTTACAAGGA
	OsHSP20−R	CCCAACAAAGGAACCAAGTT
18sRNA (AF069218)	*18SrRNA*−F	GTGGTGACGGGTGACGGAGAAT
	18SrRNA−R	CGCCTGCTGCCTTCCTTGG
