# Supplementary material for: The Promising B−Type Response Regulator hst1 Gene Provides Multiple High Temperature and Drought Stress Tolerance in Rice
Source: Int J Mol Sci. 2024 Feb 17;25(4):2385. doi: 10.3390/ijms25042385 (PMC10889171; doi:10.3390/ijms25042385)
Supplement: Supplementary file 1 [file ijms-25-02385-s001.zip › Table S3.pdf]

Table S3. Loading values and percentage contribution of variables on the axis identified by the principal component analysis (PCA) for all comparisons

Variables	Dim1			Dim2			Dim3		
	Loading values	Contribution of variable (%)	Quality of variable	Loading values	Contribution of variable (%)	Quality of variable	Loading values	Contribution of variable (%)	Quality of variable
SL	-0.831	3.566	0.690	-0.044	0.021	0.002	0.051	0.056	0.003
RL	-0.700	2.533	0.490	-0.495	2.646	0.245	0.407	3.573	0.166
SFW	-0.839	3.635	0.703	0.049	0.026	0.002	-0.092	0.182	0.008
RFW	-0.608	1.910	0.370	-0.214	0.493	0.046	-0.191	0.787	0.036
SDW	-0.770	3.061	0.592	-0.091	0.089	0.008	0.108	0.253	0.012
RDW	-0.611	1.932	0.374	0.294	0.933	0.086	0.557	6.691	0.310
RWC	-0.878	3.979	0.770	-0.067	0.048	0.004	0.037	0.030	0.001
An	0.494	1.262	0.244	-0.363	1.421	0.132	-0.303	1.986	0.092
E	0.157	0.127	0.025	0.903	8.811	0.816	-0.179	0.689	0.032
Gs	0.093	0.045	0.009	0.894	8.628	0.799	-0.113	0.274	0.013
Chla	-0.385	0.768	0.149	0.825	7.339	0.680	0.064	0.088	0.004
Chlb	0.220	0.249	0.048	0.800	6.900	0.639	0.394	3.349	0.155
ChlT	-0.192	0.190	0.037	0.886	8.476	0.785	0.193	0.802	0.037
MDA_S	0.687	2.435	0.471	-0.542	3.175	0.294	0.273	1.608	0.075
Pro_S	0.056	0.016	0.003	0.816	7.184	0.666	-0.206	0.914	0.042
SOD_S	0.024	0.003	0.001	-0.509	2.797	0.259	-0.293	1.847	0.086
APX_S	-0.467	1.127	0.218	-0.302	0.987	0.091	0.615	8.155	0.378
CAT_S	0.661	2.255	0.436	0.665	4.778	0.443	0.000	0.000	0.000
H2O2_S	0.657	2.233	0.432	-0.568	3.477	0.322	-0.444	4.258	0.197
PH_R	-0.658	2.238	0.433	0.305	1.004	0.093	0.477	4.919	0.228
PN	-0.615	1.954	0.378	0.464	2.326	0.215	-0.227	1.115	0.052
RL_R	-0.727	2.728	0.528	-0.316	1.078	0.100	0.234	1.182	0.055
RB_R	-0.809	3.379	0.654	0.182	0.359	0.033	-0.159	0.545	0.025
PB_R	-0.903	4.217	0.816	-0.173	0.322	0.030	-0.290	1.816	0.084
PL	-0.900	4.190	0.811	-0.043	0.020	0.002	0.226	1.103	0.051
SN	-0.852	3.753	0.726	-0.110	0.131	0.012	-0.177	0.674	0.031
GNPP	-0.651	2.189	0.424	-0.151	0.246	0.023	-0.541	6.309	0.292
YPP	-0.747	2.880	0.557	-0.469	2.371	0.220	-0.189	0.773	0.036
TGW	-0.679	2.381	0.461	-0.388	1.628	0.151	-0.426	3.908	0.181
MDA_R	0.597	1.842	0.356	0.372	1.496	0.139	-0.583	7.337	0.340
PRO_R	-0.622	1.999	0.387	0.303	0.993	0.092	0.018	0.007	0.000
SOD_R	0.047	0.012	0.002	0.034	0.013	0.001	0.513	5.669	0.263
APX_R	-0.548	1.553	0.301	-0.457	2.257	0.209	0.367	2.904	0.135
CAT_R	0.600	1.859	0.360	0.059	0.037	0.003	0.437	4.118	0.191
H2O2_R	0.478	1.182	0.229	0.298	0.960	0.089	0.457	4.513	0.209
An_R	0.269	0.375	0.073	-0.082	0.072	0.007	0.258	1.440	0.067
E_R	-0.004	0.000	0.000	-0.820	7.266	0.673	0.227	1.108	0.051
Gs_R	0.657	2.230	0.432	-0.098	0.103	0.010	0.531	6.092	0.282
PG	-0.890	4.096	0.793	0.324	1.131	0.105	0.020	0.009	0.000
CG	0.506	1.325	0.256	0.518	2.892	0.268	-0.321	2.226	0.103
DiG	0.888	4.070	0.788	-0.016	0.003	0.000	-0.275	1.636	0.076
DaG	0.576	1.717	0.332	-0.498	2.677	0.248	-0.296	1.892	0.088
GWdt	-0.926	4.431	0.858	0.067	0.048	0.004	-0.060	0.078	0.004
GL	-0.908	4.261	0.825	0.048	0.025	0.002	-0.278	1.671	0.077
GTh	-0.873	3.939	0.762	0.359	1.388	0.129	-0.194	0.811	0.038
Gsa	-0.866	3.873	0.750	0.293	0.925	0.086	-0.167	0.600	0.028
**Eigenvalue**		19.353			9.264			4.634	
**Variability (%)**		42.071			20.139			10.074	
**Cumulative (%)**		42.071			62.210			72.284	
